# Unusual Cortical Phenotype After Hematopoietic Stem Cell Transplantation in a Patient With Osteopetrosis

**DOI:** 10.1002/jbm4.10616

**Published:** 2022-04-29

**Authors:** Sonia Afshariyamchlou, Michelle Ng, Asmaa Ferdjallah, Stuart J. Warden, Paul Niziolek, Imranul Alam, Lynda E. Polgreen, Erik A. Imel, Paul Orchard, Michael J. Econs

**Affiliations:** ^1^ Department of Medicine Indiana University School of Medicine Indianapolis IN USA; ^2^ Department of Pediatrics University of Minnesota Medical School Minneapolis MN USA; ^3^ Department of Physical Therapy Indiana University School of Health and Human Sciences Indianapolis IN USA; ^4^ Department of Radiology Indiana University School of Medicine Indianapolis IN USA; ^5^ The Lundquist Institute at Harbor‐UCLA Medical Center Torrance CA USA; ^6^ Department of Medical and Molecular Genetics Indiana University School of Medicine Indianapolis IN USA

**Keywords:** HIGH‐RESOLUTION PERIPHERAL QUANTITATIVE COMPUTED TOMOGRAPHY (HR‐pQCT), OSTEOPETROSIS, TCIRG1, TRANSPLANTATION

## Abstract

The osteopetroses are a group of rare genetic diseases caused by osteoclast dysfunction or absence. The hallmark of osteopetrosis is generalized increased bone mineral density (BMD). However, the bone is fragile and fractures are common. Autosomal recessive osteopetrosis is usually a severe disorder and often life‐threatening in childhood. We present male siblings with autosomal recessive osteopetrosis due to biallelic variants in *TCIRG1* who survived childhood and underwent hematopoietic stem cell transplant (HSCT) in adulthood. One sibling died of posttransplant complications. After transplant, the other sibling had improvement of multiple clinical parameters, including some decline in BMD *Z*‐scores by dual‐energy X‐ray absorptiometry (DXA) and cessation of fractures. However, spine quantitative computed tomography 11 years after transplant demonstrated an anvil pattern of sclerosis with BMD *Z*‐score of +18.3. High‐resolution peripheral quantitative computed tomography (HR‐pQCT) of the tibia demonstrated near complete obliteration of the marrow space combined with an unusual cortical phenotype, suggesting extensive cortical porosity at the distal tibia. This case highlights that despite successful transplantation and subsequent improvement in clinical parameters, this patient continued to have significantly elevated bone density and decreased marrow space. Transplant‐associated increased cortical porosity is multifactorial and occurs in two‐thirds of non‐osteopetrotic patients undergoing HSCT. This finding after transplant in osteopetrosis may suggest particular sensitivity of the cortical bone to resorptive activity of transplanted osteoclasts. The case also suggests HR‐pQCT may be a useful modality for imaging and assessing the therapeutic effects on bone in individuals with osteopetrosis. © 2022 The Authors. *JBMR Plus* published by Wiley Periodicals LLC on behalf of American Society for Bone and Mineral Research.

## Graphical abstract

An adult male with autosomal recessive osteopetrosis due to variants in TCIRG1 underwent hematopoietic stem cell transplant in adulthood. After transplant, despite some decline in DXA BMD Z‐scores and cessation of fractures, spine quantitative computed tomography 11 years later demonstrated sclerosis with BMD Z‐score of +18.3. High‐resolution peripheral quantitative computed tomography (HR‐pQCT) of the tibia demonstrated in impressive cortical phenotype of high BMD, near complete obliteration of the marrow space, and extensive cortical porosity.

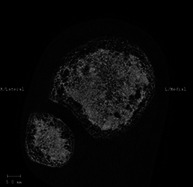

## Introduction

The osteopetroses are a group of rare genetic diseases caused by osteoclast absence or, more commonly, osteoclast dysfunction. The hallmark of osteopetrosis is generalized increased bone mineral density (BMD); however, the bone is fragile and fractures easily. Radiographic features include “rugger jersey” spine (a normal appearing vertebral midbody sandwiched between dense bone along the superior and inferior endplates) or an anvil pattern of vertebral body sclerosis, “Erlenmeyer flask deformity” (club‐shaped flaring of long bone metaphyses), and “bone‐within‐bone” or “endo bone” appearance.^(^ ^1^ ^)^ Additional clinical features can include osteomyelitis, visual loss, and bone marrow failure.^(^ ^2^, ^3^ ^)^

Autosomal recessive osteopetrosis, which is usually a severe disorder, is caused by biallelic variants in genes affecting osteoclast development (eg, *RANK*, *RANKL*) or function (eg, *TCIRG1*, *SNX10*, *CLCN7*, *OSTM1*, *CA2*, *PLEKHM1*).^(^ ^3^ ^)^ A condition originally described as type 1 autosomal dominant osteopetrosis (ADO) (now referred to as “high bone mass,” because it is not an osteopetrosis) occurs due to activating variants in *LRP5* and causes the high‐bone‐mass phenotype, but is not associated with an increase in fractures.^(^ ^4^ ^)^ Type 2 ADO is typically caused by monoallelic variants in the *CLCN7* gene.^(^ ^5^ ^)^

Severe autosomal recessive osteopetrosis has been referred to as “malignant infantile osteopetrosis”; patients typically die within the first decade of life without hematopoietic stem cell transplant (HSCT).^(^ ^6^, ^7^ ^)^ However, some cases of recessive osteopetrosis can be milder, often caused by biallelic missense variants in the above‐mentioned genes that may lead to proteins retaining some functionality. These patients typically present in childhood, but can live into early adulthood, and frequently have recurrent pathological fractures and abnormal bone morphology.^(^ ^6^ ^)^ At present, no effective medical treatment for the osteopetroses exists. HSCT is the only known therapy for autosomal recessive osteopetrosis and is typically pursued in early childhood.^(^ ^7^ ^)^

BMD is routinely assessed in patients with osteopetrosis; however, the increased areal BMD (aBMD) on dual‐energy X‐ray absorptiometry (DXA) at all skeleton sites observed in osteopetrosis does not adequately reflect bone quality or fracture risk. High‐resolution peripheral quantitative computed tomography (HR‐pQCT) provides three‐dimensional (3D) images allowing for analyses of volumetric BMD (vBMD) and bone microarchitecture with low radiation exposure. HR‐pQCT may be a useful marker for imaging individuals with osteopetrosis^(^ ^8^ ^)^ and assessing the efficacy of therapeutic candidates. Here, we present novel HR‐pQCT findings of cortical porosity (and clinical response and radiographic assessment) of a 45‐year‐old man with autosomal recessive osteopetrosis who underwent HSCT at 33 years of age.

## Patients and Methods

### Lumbar QCT

Trabecular BMD was measured in the lumbar spine by QCT at L_1_, L_2_, and L_3_, using a 64‐slice helical CT scanner (Brilliance CT 64 Channel; Philips Medical Systems, Best, the Netherlands) and QCT phantom (Image Analysis QCT‐Bone Mineral™ Phantom; Image Analysis Inc, Columbia, KY, USA). The patient was supine with the phantom beneath the patient, and CT imaging was performed with continuous axial slices from L_1_ through L_3_ based on scout images. The following CT imaging parameters were used: 120 kV, 300 mA, 2 mm slice thickness, and no dose modulation. At the center axial slice of each vertebral body, a region of interest (ROI) density measurement of each of the three components of the phantom was obtained. An elliptical ROI was also manually drawn within the vertebral body at the center axial slice of each included vertebral body. BMD for each vertebral body was subsequently derived from the calibration measurements. Data extracted from sex‐specific healthy controls from Cann and colleagues^(^ ^9^ ^)^ was then used to calculate the *Z*‐score for each vertebral body.

HR‐pQCT (XtremeCT II; Scanco Medical, Brüttisellen, Switzerland) operating at 68 kVp and 1.47 mA was used to acquire 168 tomographic slices (10.2 mm of bone length) of the distal tibia and tibial diaphysis with a voxel size of 60.7 μm. After performance of a scout view, a reference line was placed at the center of the tibia joint surface. Scan stacks were centered 7.3% and 30% of bone length proximal to the reference line. Bone length was measured using anthropometric calipers.

DXA was measured at the university of Minnesota using GE Lunar Prodigy densitometer and in Indianapolis using a Norland Elite densitometer. Biochemistries were described from the clinical record.

## Case Report

Two brothers were diagnosed with osteopetrosis in early childhood, both living into adulthood. The proband was diagnosed with autosomal recessive osteopetrosis after a hip fracture at age 3 years. The younger brother was diagnosed at age 6 months. Their family history was otherwise negative for osteopetrosis. Genetic testing of both brothers revealed biallelic variants in exon 11: NM_006019.4(TCIRG1):*c.1249G>A* (p.Ala417Thr) and exon 15: NM_006019.4(TCIRG1):*c.1735G>A* (p.Gly579Arg) of the *TCIRG1* gene.

Both of these are missense variants with minor allele frequency <0.01 and were considered as variants of uncertain significance. We analyzed these variants using three protein prediction programs: Sorting Intolerant From Tolerant (SIFT), Polymorphism Phenotyping v2 (PolyPhen‐2), and Protein Variation Effect Analyzer (PROVEAN). SIFT scores range from 0.0 (deleterious) to 1.0 (tolerated) whereas PolyPhen‐2 ranges from 0.0 (tolerated) to 1.0 (deleterious) and PROVEAN score is equal to or below a predefined threshold (score below −2.5 is considered deleterious whereas score above −2.5 is considered neutral). These programs predict the possible impact of an amino acid substitution on the structure and function of a human protein. Our analysis shows that the Ala417Thr variant is predicted to be deleterious as the SIFT score is 0.0, PolyPhen‐2 score is 0.985, and PROVEAN score is −3.596. The Gly579Arg variant is also predicted to be deleterious by the SIFT and PolyPhen‐2 programs, with scores of 0.02 and 0.824, respectively. However, the PROVEAN predicts the Gly579Arg variant is neutral as the score is −1.7. Overall, these data suggest that these substitutions likely affect protein structure/function of TCIRG1. Both variants have been reported in patients with osteopetrosis.^(^ ^10^ ^)^ The Ala417Thr variant has also been previously reported in association with risk for glioma^(^ ^11^ ^)^ and also with lower neutrophil count^(^ ^12^ ^)^; however, the Gly579Arg variant has not been reported to be associated with any other diseases. Further molecular analysis and functional data are thus necessary to fully determine the pathogenicity of these variants.

All procedures performed in studies involving human participants were in accordance with the ethical standards of the institutional research committee and with the Declaration of Helsinki and the study was approved by the Indiana University Institutional Review Board. Written informed consent was obtained from the participant.

### Patient 1

The proband went on to experience several long‐bone fractures and multiple episodes of mandible osteomyelitis. At age 37 years, he underwent an 8/8 matched unrelated bone marrow transplant. His posttransplant course was complicated by a small bowel obstruction, grade 4 graft versus host disease (GVHD) of the liver and intestines, as well as *Fusarium* fungal infection of the lungs and fungemia requiring intubation and hemodialysis. He died after an episode of sudden bradycardia 4 months after transplant.

### Patient 2

The younger brother was asymptomatic until 10 years old when he began to develop fractures. By the time he presented for evaluation for possible HSCT at age 33 years he had sustained 12 major fractures (requiring eight surgeries), including right femur shaft fractures at ages 11 and 16 years, a left humerus fracture complicated by nonunion, a right scapular fracture, right femoral intertrochanteric fracture complicated by nonunion, and total hip replacement, plus approximately 20 minor fractures. His clinical course was further complicated by a fungal skin infection at age 23 years, cholecystectomy at age 24 years, osteomyelitis of right maxilla at age 25 years requiring chronic antibiotic therapy, jaw osteonecrosis requiring debridement at age 26 years, and submental fistula and recurrent drainage from chronic osteomyelitis at age 31 years.

On physical exam at age 33 years, his height was 63 inches (*Z*‐score −2.3) and weight 166 lbs (body mass index 29 kg/m^2^). He had a large healing inner right mandible abscess, a small submental fistula, several missing teeth, and hepatosplenomegaly. His right leg was shorter than the left. His neurologic exam was normal, including intact vision and hearing.

**Fig. 1:**
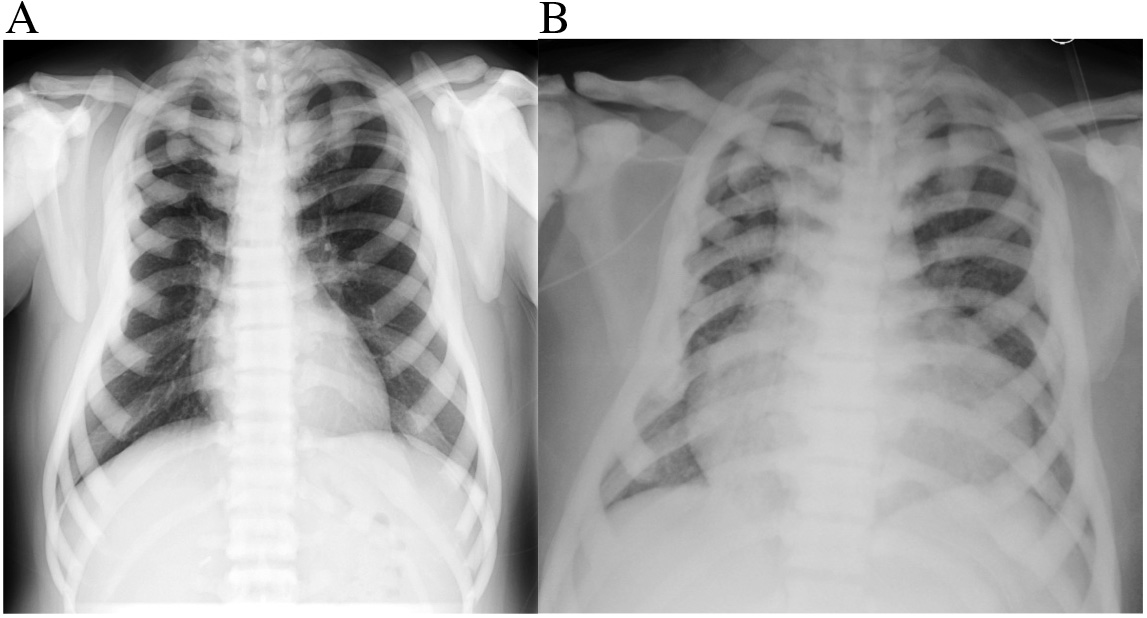
Chest radiographs before and after transplantation in adult with osteopetrosis. Chest radiographs are shown for patient 2, (*A*) before transplantation and (*B*) after transplantation, demonstrating generalized increased bone density of all bones, and a rugger Jersey spine appearance.

Laboratory evaluation revealed vitamin D deficiency, with normal alkaline phosphatase, urine collagen cross‐linked N‐telopeptide (NTX), and complete blood count (CBC). Chest radiograph did not show active pulmonary disease (Fig. 1A,B before and after transplantation, respectively), but pulmonary function testing showed mildly reduced diffusion capacity for carbon monoxide (DLCO) (54% predicted). Echocardiogram was normal with LVEF: 55%–60%. Total body BMD *Z*‐score measured by DXA was +12.4 (Table 1).

**Table 1:** Bone Mineral Density and Biochemistry Changes After HSCT

Parameter	Before transplant	Posttransplant				
Age (years)	33	34	35	37	40	45
Location	UMN	UMN	UMN	UMN	UMN	IU
DXA machine	GE Lunar Prodigy					Norland Elite
*Z*‐scores						
Total body	12.4	13.1	12.1	11.8	10	
Lumbar spine	14.6	15.2	11.1	15.6	14.3	14.6
Femur neck	13.4	13.1	13.0	13.1	10.2	15.5
Total hip	12.4	13.1	12.1	11.8	10.0	13.6
Alkaline phosphatase (40–150 U/L)	46	Values ranging from 142–1539	272	94	106	
Bone alkaline phosphatase (6–20 U/L)		15.2	25.8	11.6	11.2	
Osteocalcin (11–50 ng/mL)				27	21	
CTX (70–780 pg/mL)		951				
Urine NTX (21–83 BCE/mM)	60			83		

Abbreviations: IU, Indiana University; UMN, University of Minnesota.

He underwent an HSCT with an 8/8 matched unrelated donor at age 33 years achieving donor engraftment, following a reduced intensity conditioning regimen consisting of alemtuzumab, busulfan, fludarabine, and total body irradiation. He maintained 100% donor chimerism following his allogeneic transplant. Immediate posttransplant complications included hypercalcemia, sensorineural hearing loss, acute GVHD, *Candida* sepsis, and multiorgan dysfunction.

One year after HSCT, his submental fistula healed, he underwent mandibular bone grafting, and his serum C‐telopeptide (CTX) as a marker of bone resorption was mildly increased at 951 pg/mL [normal 70–780]. His total body BMD *Z*‐score had increased from +12.4 to +13.1.

Over the next several years complications included chronic GVHD affecting liver, skin, and mouth treated with sirolimus, along with fungal infections and pericardial effusion. He required multiple treatment modalities for his GVHD including prolonged therapy with steroids, sirolimus and photopheresis. Four years after HSCT, markers of bone resorption (NTX) and bone formation (osteocalcin, bone‐specific alkaline phosphatase) were normal and total body BMD *Z*‐score decreased to +11.8. Seven years after HSCT, total body BMD *Z*‐score decreased further to +10.0.

He was evaluated at Indiana University as part of a natural history study at 11 years posttransplant. He complained of chronic mucus membrane problems after HSCT. He also complained of fatigue and mild bone and joint pain. He was taking doxycycline as prophylaxis for osteomyelitis of the maxilla. On physical exam he appeared to be chronically ill. He had multiple missing teeth, and mucosal candidiasis in his mouth. He had chronic skin changes since HSCT including multiple hyperpigmented macules on his skin. Musculoskeletal exam was significant for multiple deformities, which were complications of past fractures. However, he had not had any fractures in the 11 years since transplant.

**Fig. 2:**
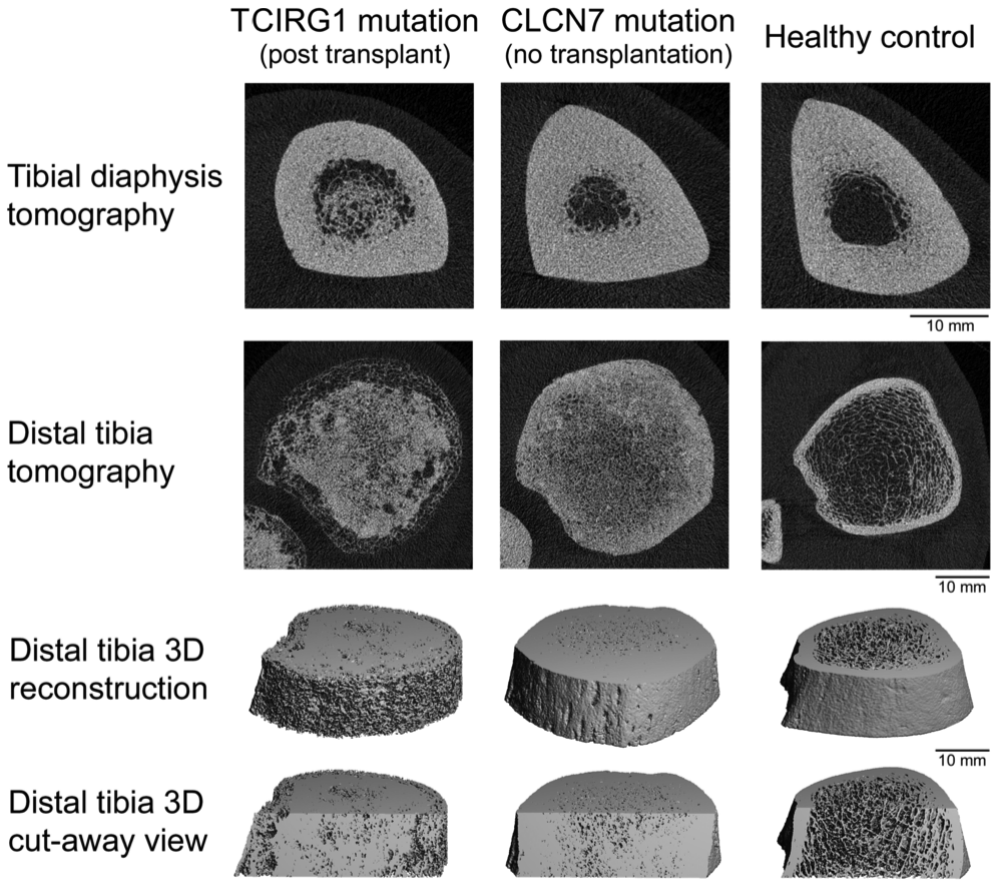
High‐resolution peripheral quantitative computed tomography images of the patient's distal tibia. 2D and 3D tomographic views and frontal cut‐away view show near complete obliteration of the trabecular compartment and extensive cortical porosity in patient 2, 11 years posttransplant (left images). For comparison, an adult male with autosomal dominant osteopetrosis due to CLCN7 mutation (middle images) and a healthy control adult male (right images) are shown.

His spine QCT indicated an anvil pattern of sclerosis with *Z*‐score +18.3. HRpQCT measurements of his tibia indicated an unusual pattern of near complete obliteration of the marrow space; however, there is an unusual cortical phenotype, suggesting extensive cortical porosity (Fig. 2). To the best of our knowledge, this is the first report of HRpQCT in an osteopetrosis patient posttransplant. It will be important to repeat these measurements in additional patients to determine the frequency of this finding.

## Discussion

Autosomal recessive osteopetrosis is typically life‐threatening in childhood complicated by fractures and bone marrow failure, and the only treatment currently available is HSCT. In comparison with patients with the severe form of autosomal recessive disease, the indication for HSCT in patients with attenuated recessive disease is less clear. This patient is unusual in that he survived into adulthood before requiring treatment with HSCT. After HSCT, he stopped fracturing and had other clinical improvements, though he also suffered severe acute and chronic complications. Stepensky and colleagues^(^ ^7^ ^)^ reported that seven patients with attenuated autosomal recessive osteopetrosis, two of whom had *TCIRG1* variants, had significant improvement in symptoms and quality of life after HSCT. It was suggested that patients with attenuated forms of autosomal recessive osteopetrosis should be considered for HSCT. However, it should also be acknowledged that the complications of HSCT can be significant, including death, as was seen in the proband in our report. Additionally, it is unknown whether those with ADO (due to *CLCN7* variants) benefit from HSCT.

We evaluated an adult patient with autosomal recessive osteopetrosis due to *TCIRG1* variants by DXA and HRpQCT 11 years after HSCT. Although measured on different densitometers between two institutions, the bone density *Z*‐scores on DXA did decrease after HSCT, at least at the spine and total body. HR‐pQCT findings demonstrated an unusual cortical phenotype, which may be consistent with extensive cortical porosity, possibly due to bone resorption in the cortex. Of note, the CTX was mildly elevated after transplant and the NTX was at the upper limits of normal, consistent with increased osteoclastic resorption after transplant. This patient had numerous reasons to develop increased cortical porosity. Transplant‐associated increased cortical porosity is multifactorial and occurs in two‐thirds of patients undergoing HSCT.^(^ ^13^ ^)^ Total body irradiation can certainly lead to increased cortical porosity as can its secondary effects such as hypogonadism. This patient required years‐long therapy with glucocorticoids, which are classically associated with poor bone density.^(^ ^14^ ^)^ Cytotoxic agents, including the busulfan he received as part of his conditioning therapy, are also associated with bone loss.^(^ ^15^ ^)^

Detailed evaluation of recessive or dominant osteopetrosis patients with HR‐pQCT are scarce in the literature. A case report of two individuals with ADO2, HR‐pQCT found increased density, increased cortical thickness, high trabecular number and thickness, and increased bone volume fraction (BV/TV).^(^ ^8^ ^)^ However, the most prominent feature was the great heterogeneity in bone microstructure at visual analyses of the multiple CT slices of the affected patients. Islets of very dense bone were found interposed with areas with apparent normal density along all the slices evaluated by HRpQCT.^(^ ^1^ ^)^ This heterogeneity, which reflects defective bone resorption and areas of “bone inside bone,” may contribute to bone fragility and fractures in patients with osteopetrosis. Unfortunately, there are no other reports of HR‐pQCT data from osteopetrosis patients post‐HSCT. It will be important to confirm if the unusual cortical phenotype, seen in this patient, is typical for patients who receive HSCT either during childhood or as adults.

This case report highlights both the complications and effectiveness of HSCT in an adult with autosomal recessive osteopetrosis. Clinically there was improvement in mandibular osteomyelitis and decrease in fracture rate, along with declines in *Z*‐scores on DXA. Although HSCT did not cure the osteopetrosis (QCT *Z*‐score +18.3, and DXA *Z*‐scores +13.6 to +15.5), he has had other clinical benefits from the transplant such as cessation of fractures, improving bone pain and osteomyelitis.

The HR‐pQCT illustrates the persistent abnormal trabecular bone microarchitecture combined with an abnormal extensive cortical phenotype post‐HSCT in a patient with autosomal recessive osteopetrosis. HR‐pQCT may be a useful marker for following therapeutic effectiveness in osteopetrosis.

## Disclosure

MJE, EAI, and LEP report past research funding by Horizon Pharmaceuticals. All other authors report no conflicts of interest related to this work.

### Peer Review

The peer review history for this article is available at https://publons.com/publon/10.1002/jbm4.10616.

## References

[1] Ladd LM , Imel EA , Niziolek PJ , et al. Radiographic imaging, densitometry and disease severity in autosomal dominant osteopetrosis type 2. Skeletal Radiol. 2021;50(5):903‐913. 33009917 10.1007/s00256-020-03625-3 PMC8009803

[2] Benichou OD , Laredo JD , de Vernejoul MC . Type II autosomal dominant osteopetrosis (Albers‐Schonberg disease): clinical and radiological manifestations in 42 patients. Bone. 2000;26(1):87‐93. 10617161 10.1016/s8756-3282(99)00244-6

[3] Sobacchi C , Schulz A , Coxon FP , Villa A , Helfrich MH . Osteopetrosis: genetics, treatment and new insights into osteoclast function. Nat Rev Endocrinol. 2013;9(9):522‐536. 23877423 10.1038/nrendo.2013.137

[4] Van Wesenbeeck L , Cleiren E , Gram J , et al. Six novel missense mutations in the LDL receptor‐related protein 5 (LRP5) gene in different conditions with an increased bone density. Am J Hum Genet. 2003;72(3):763‐771. 12579474 10.1086/368277 PMC1180253

[5] Waguespack SG , Koller DL , White KE , et al. Chloride channel 7 (ClCN7) gene mutations and autosomal dominant osteopetrosis, type II. J Bone Miner Res. 2003;18(8):1513‐1518. 12929941 10.1359/jbmr.2003.18.8.1513

[6] Wu CC , Econs MJ , DiMeglio LA , et al. Diagnosis and management of osteopetrosis: consensus guidelines from the Osteopetrosis Working Group. J Clin Endocrinol Metab. 2017;102(9):3111‐3123. 28655174 10.1210/jc.2017-01127

[7] Stepensky P , Grisariu S , Avni B , et al. Stem cell transplantation for osteopetrosis in patients beyond the age of 5 years. Blood Adv. 2019;3(6):862‐868. 30885997 10.1182/bloodadvances.2018025890 PMC6436016

[8] Arruda M , Coelho MC , Moraes AB , et al. Bone mineral density and microarchitecture in patients with autosomal dominant Osteopetrosis: a report of two cases. J Bone Miner Res. 2016;31(3):657‐662. 26387875 10.1002/jbmr.2715

[9] Cann CE , Genant HK , Kolb FO , Ettinger B . Quantitative computed tomography for prediction of vertebral fracture risk. Bone. 1985;6(1):1‐7. 3994856 10.1016/8756-3282(85)90399-0

[10] Pangrazio A , Caldana ME , Lo Iacono N , et al. Autosomal recessive osteopetrosis: report of 41 novel mutations in the TCIRG1 gene and diagnostic implications. Osteoporos Int. 2012;23(11):2713‐2718. 22231430 10.1007/s00198-011-1878-5

[11] Kinnersley B , Kamatani Y , Labussiere M , et al. Search for new loci and low‐frequency variants influencing glioma risk by exome‐array analysis. Eur J Hum Genet. 2016;24(5):717‐724. 26264438 10.1038/ejhg.2015.170 PMC4677454

[12] Rosenthal EA , Makaryan V , Burt AA , et al. Association between absolute neutrophil count and variation at TCIRG1: the NHLBI Exome Sequencing Project. Genet Epidemiol. 2016;40(6):470‐474. 27229898 10.1002/gepi.21976 PMC5079157

[13] Kelly PJ , Atkinson K , Ward RL , Sambrook PN , Biggs JC , Eisman JA . Reduced bone mineral density in men and women with allogeneic bone marrow transplantation. Transplantation. 1990;50(5):881‐883. 2238065

[14] Canalis E , Giustina A . Glucocorticoid‐induced osteoporosis: summary of a workshop. J Clin Endocrinol Metab. 2001;86(12):5681‐5685. 11739419 10.1210/jcem.86.12.8066

[15] Pandit A , Garg MK , Kotwal N , et al. Changes in bone mineral density and bone turnover markers in patients undergoing hematopoietic stem cell transplant. Indian J Endocrinol Metab. 2015;19(3):393‐398. 25932397 10.4103/2230-8210.152785 PMC4366780

